# Context-dependent expression of the *foraging* gene in field colonies of ants: the interacting roles of age, environment and task

**DOI:** 10.1098/rspb.2016.0841

**Published:** 2016-08-31

**Authors:** Krista K. Ingram, Deborah M. Gordon, Daniel A. Friedman, Michael Greene, John Kahler, Swetha Peteru

**Affiliations:** 1 Department of Biology, Colgate University, 13 Oak Drive, Hamilton, NY 13346, USA; 2 Department of Biology, Stanford University, Gilbert Biological Science Building, Stanford, CA 94305, USA; 3 Department of Integrative Biology, University of Colorado, Campus Box 171, PO Box 176634, Denver, CO 80217-3364, USA; 4 Department of Geography, Texas A&M University, College Station, TX 77843, USA

**Keywords:** *foraging* gene, social insect, task allocation, division of labour

## Abstract

Task allocation among social insect workers is an ideal framework for studying the molecular mechanisms underlying behavioural plasticity because workers of similar genotype adopt different behavioural phenotypes. Elegant laboratory studies have pioneered this effort, but field studies involving the genetic regulation of task allocation are rare. Here, we investigate the expression of the *foraging* gene in harvester ant workers from five age- and task-related groups in a natural population, and we experimentally test how exposure to light affects *foraging* expression in brood workers and foragers. Results from our field study show that the regulation of the *foraging* gene in harvester ants occurs at two time scales: levels of *foraging* mRNA are associated with ontogenetic changes over weeks in worker age, location and task, and there are significant daily oscillations in *foraging* expression in foragers. The temporal dissection of *foraging* expression reveals that gene expression changes in foragers occur across a scale of hours and the level of expression is predicted by activity rhythms: foragers have high levels of *foraging* mRNA during daylight hours when they are most active outside the nests. In the experimental study, we find complex interactions in *foraging* expression between task behaviour and light exposure. Oscillations occur in foragers following experimental exposure to 13 L : 11 D (LD) conditions, but not in brood workers under similar conditions. No significant differences were seen in *foraging* expression over time in either task in 24 h dark (DD) conditions. Interestingly, the expression of *foraging* in both undisturbed field and experimentally treated foragers is also significantly correlated with the expression of the circadian clock gene, *cycle*. Our results provide evidence that the regulation of this gene is context-dependent and associated with both ontogenetic and daily behavioural plasticity in field colonies of harvester ants. Our results underscore the importance of assaying temporal patterns in behavioural gene expression and suggest that gene regulation is an integral mechanism associated with behavioural plasticity in harvester ants.

## 1. Introduction

One of the most exciting new frontiers in sociogenomics is investigating how behavioural plasticity in advanced social organisms is regulated by molecular mechanisms [1–7]. Social insects provide an ideal system for studying the evolution and ecology of behavioural plasticity because the ecological success of a colony depends on task allocation [2,3,8,9]. Colonies operate without central control. Individuals respond to local cues; and in the aggregate, the colony adjusts the numbers of workers performing various tasks, in response to current conditions [10]. Recent molecular studies present evidence for strong links between differential gene regulation and worker development [11,12], behaviour [7,13–16] and social environment [13,15,17,18]. These studies, in conjunction with the sequencing of many social insect genomes [19–27], provide the critical groundwork for detailed functional analysis of target genes and their effect on social insect behaviour.

Task allocation differs among social insect species. In many social insect societies, workers progress through tasks in an age-dependent manner, a process termed temporal polyethism [28]. Diverse mechanisms, using conserved molecular pathways, interact to regulate age-polyethism in workers [3]. For example, genetic pathways involved in nutrition and metabolism play a major role in the regulation of worker task (reviewed in [3,29]). Causal relationships between gene regulation and age-related transitions in worker task have been documented for *malvolio*, a gene involved in manganese transfer and sucrose responsiveness [30], the storage protein gene, *vitellogenin* [31], the insulin-signalling TOR pathway [32,33] and *foraging*, a cyclic GMP-activated protein kinase [34–36]. The results from these studies highlight the complexity of relationships between conserved genetic pathways and transitions to foraging in social insects.

The *foraging* gene, a cGMP-activated protein kinase gene (PKG), has emerged as a behavioural gene of particular interest due to the diversity of relationships between the expression of this gene and behaviour [14,29,34,37–45]. *Foraging* is associated with behaviour in diverse taxa including nematodes, insects and mammals [40,44]. However, associations between this gene and behaviour vary across species in both mechanism and proposed function, ranging from learning and memory to chemotaxis and food-related behaviours [40,44]. PKG is activated by a common secondary messenger (cGMP) and, when activated, phosphorylates a host of cellular proteins [46]. Thus, this gene is associated with a diverse range of behavioural and physiological processes [44].

The *foraging* gene was originally described in fruit flies [37] and has been shown to have a direct link to foraging behaviour in several insect species [34,35,37,43]. *foraging* has also been shown to influence habituation and sucrose responsiveness, stress tolerance, olfactory and visual learning, memory and sleep patterns in fruit flies [47–49].

In social insects, the *foraging* gene is implicated in the age-related transition from other tasks to foraging [34,35,39,42,43,50]. In honeybees, *Polistes metricus* wasps and *Bombus terrestris* bumblebees, foragers have higher levels of expression of *foraging* than nurse bees [32,34,50]. By contrast, studies on *Vespula* wasps, *Bombus ignites* bumblebees and harvester ants suggest that workers that forage have lower levels of *foraging* mRNA than workers that do not forage [39,42,43,51]. Similarly, the ant *Pheidole pallidula* shows high activity of this gene in the soldier caste and low expression in minor workers that engage frequently in foraging [43]. Interestingly, an experimental manipulation of same-age cohorts and tasks in *Cardiocondyla obscurior* demonstrated that *foraging* expression in this short-lived ant is correlated with age, but not with the foraging task [52].

Here, we explore *foraging* gene expression and task allocation in a natural population of red harvester ants (*Pogonomyrmex barbatus*). In previous work, we showed that the expression of a harvester ant orthologue (*Pbfor*) to *foraging* at dawn was lower in foragers than workers of other tasks, including brood care (nurse) workers [39]. Harvester ants live in large colonies of up to 12 000 workers in the southwestern deserts of the United States [53]. Temporal polyethism in harvester ants occurs over the course of a year, the approximate lifespan of a worker [54,55]. Younger workers perform tasks related to brood care and do not leave the nest. Workers then progress to nest maintenance work, with brief trips out of the nest to carry out refuse, then to patrolling, with short morning forays from the nest, and finally to foraging [56–58]. Foragers spend the most time out of the nest, leaving in early morning and foraging until mid-afternoon.

In the field, foraging activity occurs in a daily temporal pattern [59,60]. The discovery of task-specific expression of circadian clock genes in harvester ants confirmed that foragers have a functional molecular clock and endogeneous circadian rhythms, while workers that perform tasks inside the nest do not show pronounced circadian rhythms in activity levels or expression of clock genes [61]. In addition, results from a laboratory study on *Pogonomyrmex occidentalis*, a congener of *P. barbatus,* revealed that the expression of the *foraging* gene in workers can vary with time of day [62]. Foragers of *P. occidentalis* had low levels of *foraging* mRNA only during late evening and early morning hours, and had high levels of *foraging* mRNA relative to non-foraging workers during the daytime. These laboratory results led us to question whether the previous finding of low *foraging* gene expression in *P. barbatus* foragers was influenced by the early morning collection time of the field samples.

Here, we explore how gene expression correlates with the temporal regulation of foraging behaviour in a natural population of harvester ants. By investigating gene expression as it occurs in the field, we are able to investigate the molecular responses to the natural cues of temperature, light and interactions among workers [63–66] that influence the circadian pattern of foraging activity. We consider two time scales, asking how *foraging* expression is associated with daily individual activity rhythms during a circadian cycle, and how the patterns of expression are associated with task transitions over weeks to months as workers mature. In addition, we experimentally manipulate the light conditions of field-collected brood workers and foragers to test whether the expression of *foraging* is correlated with exposure to light and the expression of the circadian clock gene, *cycle*.

## 2. Material and methods

### (a) Ant collection

Workers of *P. barbatus* were collected from colonies near our long-term study site in Rodeo, New Mexico. To ensure representation of all behavioural tasks and to replicate findings across colonies, workers were collected from large, mature colonies in the early morning hours (*n* = 4 colonies for field study, *n* = 6 colonies for experimental study). Workers were designated as belonging to one of five groups: callows, brood workers, nest maintenance, patrollers or foragers, defined as in previous work [58,60]. We considered three age-related worker categories: immature workers (called callows) and two of the task groups (brood care workers and foragers) which represent clear transitions in the maturation of workers. Two other tasks (patrolling and nest maintenance) are more labile, so are less tightly associated with worker age [57,58]. Patrollers, foragers and, to a lesser extent, nest maintenance workers are exposed to external environmental conditions [67].

Patrollers, which were the first group we collected in the morning, emerge at first light and travel around the nest mound and foraging area, often with the abdomen tucked under the thorax [68]. Patrollers stimulate the start of foraging upon returning to the nest and influence the direction of foraging each day. Nest maintenance workers make short trips outside the nest to carry out debris and discard it in a pile away from the nest entrance. Newly emerged adults (callows) are identified by their distinct orange-coloured exoskeleton. In laboratory colonies, callows are usually found near the brood and queen, and may participate in brood work [58]. Brood care workers are young workers that are found inside brood chambers. We collected only brood care workers that were carrying brood in their mandibles. Foragers were collected in late morning, while they were returning to the nest carrying food. The nests were then excavated to collect the brood workers and callows.

### (b) Field experiment set-up for time of day sampling

Callows and brood workers were placed together in 30.5 × 30.5 cm plastic boxes with sand and brood in complete darkness. Nest maintenance and patrollers were placed in plastic boxes with sand and rocks that were dark on one side and exposed to ambient light on the other side; foragers were placed in plastic boxes with sand and grasses, and half of the box was exposed to ambient light. The boxes were housed in ambient temperature conditions in a laboratory at the Southwestern Research Station (American Museum of Natural History) in Portal, Arizona. To control for potential effects of alarm responses induced during the transport of ants to the laboratory, we made certain that all task groups were exposed to the same handling conditions. In the course of the subsequent 24 h, a sample of 4–5 ants from each task and colony were removed at 4 h intervals, and flash frozen in liquid nitrogen. All sampling during evening hours was done using dim red light in dark conditions. Ants were collected from each colony at seven time points: 16.00, 20.00, 24.00, 4.00, 8.00, 12.00 and 16.00 (day 2). Approximate daylight hours during sampling were 13 h of daylight (5.30–18.30). Frozen ants were stored in the −80°C freezer prior to brain dissection.

### (c) Light exposure experiment

Foragers and brood workers (*n* = 18 workers per task per colony) were collected from six medium-sized field colonies as described above. Workers were immediately placed into artificial nest-boxes (*n* = 24 nest-boxes in total) in one of two treatments: 13 L : 11 D (LD) ants were placed in nest-boxes (with water ad libitum) with ambient daytime light and night-time darkness; DD ants were housed in identical nest-boxes in complete darkness for 24 h. Nest-boxes with inside workers contained some dirt/sand and brood from the original colony. Forager nest-boxes contained some dirt/sand and local leaves. Workers were sampled from these artificial nest-boxes at three time points (13.00, 21.00 and 5.00). Red lights were used to sample during the dark hours to minimize light exposure. Live workers were placed immediately into cryovials and then flash frozen in liquid nitrogen. Frozen samples were stored on dry ice, shipped to Colgate University and frozen at minus 80°C prior to brain dissection.

### (d) RNA extraction and quantitative real-time PCR

Brains were dissected on dry ice and placed immediately into RNAlater (Ambion) to remove glands. Whole brains (including optic lobes) were placed immediately in lysis buffer and homogenized with Qiashedders (Qiagen). RNA was purified from three homogenized brains per sample using RNAeasy Micro Kit (Qiagen) protocols. Purified RNA was frozen at 80°C prior to qPCR procedures. Harvester ant-specific primers for qPCR analyses were designed from exon-coding regions to amplify a 128 bp region of *foraging* using the newly sequenced genome [22,23]. cDNA was synthesized from extracted total RNA preps using ABI TaqMan Gold Reverse Transcriptase reagents and random hexamers. The 10 µl reactions included 1.0 µl of RNA with 1× TaqMan RT Buffer, 5.5 mM MgCl_2_, 500 uM of each of the deoxyNTPs, 2.5 uM of the random hexamer primers, 0.4 U µl^−1^ of RNase Inhibitor and 1.3 U µl^−1^ of MultiScribe Reverse Transcriptase (50 U µl^−1^). Each colony had one sample (*n* = 3 brains) per time point; reactions were performed in triplicate for each sample (*n* = 3 technical replicates). All reactions were run at 25°C for 10 min, 48°C for 30 min and 95°C for 5 min, and then stored at −20°C until quantitative PCR. For each cDNA replicate, expression of *Pbfor* was assayed on an ABI 7900 HT instrument using ABI Taqman Gold reagents and primers designed as a Taqman Gene Expression Assay (table 1). The 25 µl qPCR reactions for *foraging* included 3.5 µl of template cDNA with 1× TaqMan Buffer A, 5.5 mM MgCl_2_, 200 µM each of dNTPs, 100 nM of probe, 200 nM of each primer, 0.01 U µl^−1^ of AmpErase UNG and 0.025 U µl^−1^ of AmpliTaq Gold DNA Polymerase (50 U µl^−1^). To standardize *foraging* expression, *elongation factor 1α* (64 bp) was used as a control for each cDNA replicate. Of the three control genes tested (*PbEF1α*, *Pb18S* and *PbRPII*), the amplification efficiency of *PbEF1α* was most similar to the *foraging* gene and levels of *PbEF1α* did not vary over time. The 25 µl qPCR reactions for the control included 1.5 µl of template cDNA. For the light exposure experiment, expression of the *cycle* gene was also measured with the same procedure used for *foraging* expression.

**Table 1.** qPCR primers designed for study.

gene	forward primer	reverse primer	probe
*PbFor*	TGGTGGTGACCCAATGAAGACGTA	GTTCCGCGGGATTATCTCTG	TCCATCACGCGTAACGCAATGGCT
*PbCYCix*	GCGATATGCAGGTGAAAGAAGA	ATCACGCAATACTTCCAATCTATGTT	CGACACCACCATTGGCTGTCACAGA
*PbEF1α*	GGCTCTGAGGGAGGCTTT	CGGAGATGTTCTTCACGTTGAA	CTCGCGATAACGTCG

Real-time PCR reactions for *Pbfor* and *PbEF1α* were performed under the following conditions: 2 min at 50°C for one cycle, 10 min at 95°C for one cycle, 15 s at 95°C, 1 min at 58°C, for 45 cycles. Data were analysed using SDS 2.1 software and quantification of relative mRNA levels was calculated using the ΔΔCt method. For the field experiment, relative expression levels within colonies were calculated across all tasks and time points, and then normalized (due to potential differences in gene expression levels across colonies) using a *z*-score transformation. For both treatments in the light experiment, relative expression levels within colonies were calculated across both foraging and brood care tasks for comparisons of overall *foraging* expression between tasks. Relative gene expression was also calculated within tasks in each treatment to compare changes in expression over time for each task.

### (e) Statistical analysis

To test for differences in individual brain expression levels among groups, we tested for normality using Shapiro–Wilk tests in SPSS. Because the data did not deviate significantly from a normal distribution, we used mixed-model ANOVAs with time as the ‘within subject’ fixed factor, task, location or age as ‘between subjects' fixed factors, and colony as a random factor. For the field study, we tested three hypotheses. We tested whether *foraging* gene expression was associated with task by comparing the five behavioural tasks. We tested whether *foraging* gene expression was associated with worker environment by comparing internal workers (callows and brood care workers) to external workers (nest maintenance, patrollers and foragers). We tested whether *foraging* gene expression was associated with worker age by comparing callows (newly emerged), brood care workers (young workers) and foragers (old workers).

Differences in the pattern of relative *Pbfor* expression over time were analysed for individual task groups using mixed-model ANOVAs. We also tested the pattern of *Pbfor* expression in foragers using repeated measures contrast analyses [69]. Contrast analysis tests specific, theoretically driven, *a priori* predictions about patterns in repeated measures data. We tested the prediction that daily fluctuations in *foraging* gene expression follow observed daily rhythms in task behaviour. The expression pattern of foragers was compared with a generalized sinusoidal curve that approximates the daily foraging activity rhythms of harvester ant foragers in the field and the locomotor activity of foragers in laboratory colonies. We used Pearson's correlation analyses to test the correlation of *foraging* and *cycle* expression within each task.

To test for differences in *foraging* expression in the light exposure experiment, we used mixed-model ANOVAs with time as the ‘within subject’ fixed factor, light condition as the ‘between subjects’ fixed factor and colony as a random factor. We tested within-task differences in expression over time of day with one-way ANOVAs. We used Pearson's correlation analyses to test the correlation of *foraging* and *cycle* expression in foragers in LD and DD conditions. All analyses were performed in SPSS and we controlled for multiple testing using Bonferroni corrections.

## 3. Results

### (a) Field study

**Figure 1.**
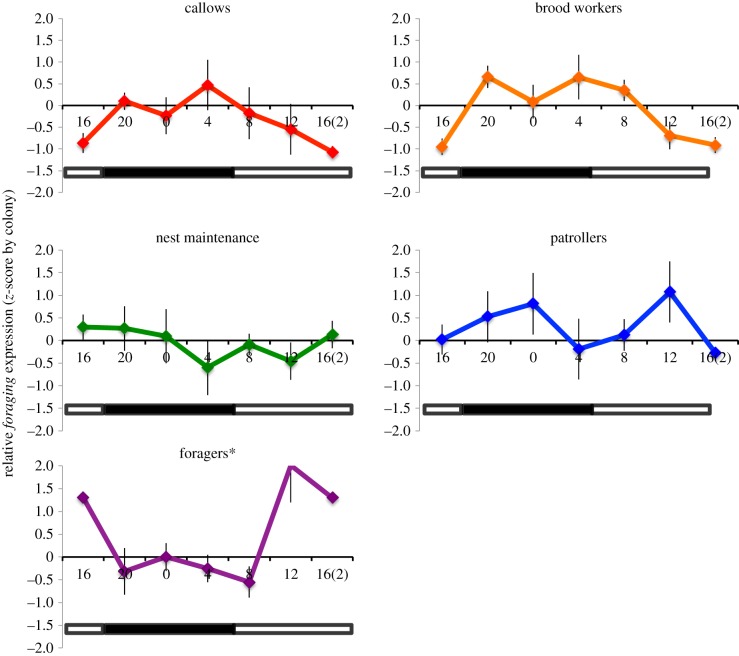
Relative gene expression levels of *foraging* across time (hours) from field-collected workers of five behavioural tasks. Relative expression values for each data point represent the average expression level across colonies (*n* = 4 colonies, ±s.e.). Data were normalized to account for differences in the amplitude of gene expression between colonies using a *z*-score transformation; thus, relative expression values are plotted as the number of standard deviations above and below the mean value for all data points (across task and time). Standard error bars are calculated from variation across four colonies. The open stripe in the horizontal bar at base of the plot represents the daylight phase (13 h) and the solid stripe represents the dark phase (11 h) during the night. Overall differences in *foraging* gene expression among tasks are significant; only foragers have significantly different levels of *Pbfor* mRNA over time. *p < 0.05. (Online version in colour.)

When considering all five tasks, gene expression varies significantly among tasks (*F*_4,98_ = 3.732, *p*_corr_ = 0.021, *η*^2^ = 0.132; figure 1 and table 2), and the interaction between task and time was significant (*F*_2,98_ = 2.521, *p*_corr_ = 0.003, *η*^2^ = 0.09). Comparisons of internal and external tasks (callows and brood care workers versus nest maintenance workers, patrollers and foragers) revealed significant differences in gene expression between locations (*F*_1,118_ = 7.895, *p*_corr_ = 0.006, *η*^2^ = 0.062), and the interaction between location and time was significant (*F*_6,118_ = 4.943, *p*_corr_ = 0.000, *η*^2^ = 0.201). When considering only age-related categories (young callows and brood care workers versus older foragers), gene expression varied significantly across age (*F*_2,62_ = 14.277, *p*_corr_ = 0.000, *η*^2^ = 0.315) and the interaction between age and time was significant (*F*_6,62_ = 9.899, *p*_corr_ = 0.000, *η*^2^ = 0.489).

**Table 2.** Mixed-model ANOVA results for field study. We tested three hypotheses: the effect of task, location or age and time of day on *foraging* expression in workers.

		d.f.	*F*	*p*-values^a^	*η*^2^
task	task	4,98	3.732	0.021*	0.132
	time	6,98	0.699	0.651	0.041
	task × time	24,98	2.521	0.003**	0.090
location	location	1,118	7.895	0.006**	0.062
	time	6,118	1.010	0.422	0.049
	location × time	6,118	4.943	0.000*	0.201
age	age	2,62	14.277	0.000**	0.315
	time	6,62	1.475	0.201	0.125
	age × time	6,62	9.899	0.000**	0.489

^a^Bonferroni-corrected *p*-values; *p < 0.05, **p < 0.01.

Foragers have significant changes in *foraging* gene expression over time (*F*_6,20_ = 3.613, *p*_corr_ = 0.019, *η*^2^ = 0.520; other tasks, see electronic supplementary material, table S1). The pattern of expression of *Pbfor* mRNA in forager brains is correlated with the generalized sinusoidal function curve predicted from the daily fluctuations in foraging behaviour (*t* = 2.78, *r* = 0.89, *p* = 0.05). Expression levels of *Pbfor* were significantly correlated with *cycle* expression in foragers only (foragers: Pearson's correlation = 0.80, *p* = 0.03; other tasks, see electronic supplementary material, table S2).

### (b) Light exposure experiment

**Figure 2.**
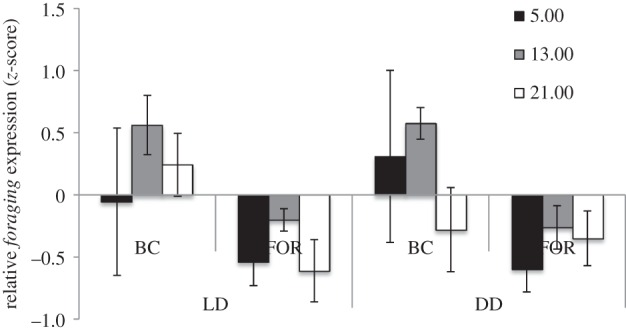
Results from the light exposure experiment. Gene expression data were measured across all samples and were transformed using a *z*-score analysis across colonies (*n* = 6) to control for differences in expression levels between colonies. Bars represent the number of standard deviations above or below the mean colony expression value (±s.e.). Differences between tasks are significant; there is no significant effect of light condition or task × light interaction on expression levels. BC = brood care workers; FOR = foragers; LD = 13 h ambient light exposure, 11 h dark; DD = continuous 24 h dark.

In the exposure experiment, we found significant differences in relative gene expression for task (*F*_1,57_ = 9.141, *p*_corr_ = 0.004, *η*^2^ = 0.138), but not for light condition (*F*_1,57_ = 0.058, *p*_corr_ = 0.810, *η*^2^ = 0.001), time of day (*F*_1,57_ = 1.643, *p*_corr_ = 0.202, *η*^2^ = 0.055) or the interaction between task and light condition (*F*_1,57_ = 0.011, *p*_corr_ = 0.916, *η*^2^ = 0.000; figure 2 and table 3).

**Table 3.** Mixed-model ANOVA results for experimental study. We tested one hypothesis: the effects of light condition (LD or DD) and time of day on *foraging* expression in workers.

	d.f.	*F*	*p*-values	*η*^2^
task	1,57	9.141	0.004**	0.138
light condition	1,57	0.058	0.810	0.001
time	2,57	1.643	0.202	0.055
task × light	1,57	0.011	0.916	0.000
task × time	2,57	0.558	0.576	0.019
light × time	2,57	0.401	0.671	0.014
task × light × time	2,57	0.585	0.560	0.020

**p < 0.01.

**Figure 3.**
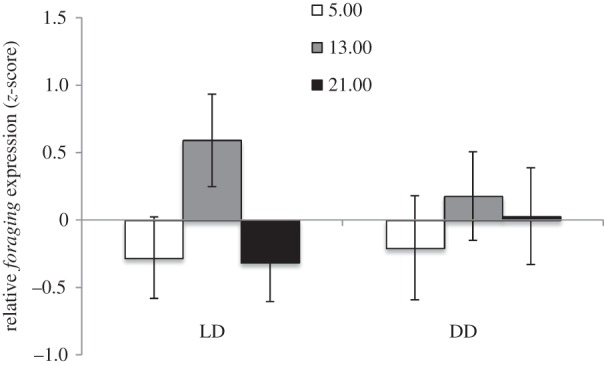
Expression levels of *foraging* mRNA differ with time of day in LD but not DD conditions in foragers. Bars represent relative gene expression calculated across foragers only. Data were transformed using a *z*-score analysis across colonies (*n* = 6) to control for differences in expression levels between colonies. Relative expression values are plotted as the number of standard deviations above and below the mean colony value for foragers (±s.e.). LD = 13 h ambient light exposure, 11 h dark; DD = continuous 24 h dark.

When relative expression is calculated within tasks across time of day, foragers differ in *foraging* levels depending on time of day only in the LD treatment, but this difference is not significant following correction for multiple tests (LD: *F*_2,9_ = 2.859, *p*_corr_ = 0.109, *η*^2^ = 0.389; DD: *F*_2,10_ = 0.982, *p*_corr_ = 0.408, *η*^2^ = 0.164; figure 3). *Foraging* expression in foragers is significantly correlated with the expression of *cycle*, a circadian clock gene in LD (Pearson's correlation = 0.627, *p* = 0.007) and DD (Pearson's correlation = 0.633, *p* = 0.005) conditions. Brood workers do not show differences in *foraging* expression depending on time of day in either treatment (LD: *F*_2,9_ = 0.670, *p*_corr_ = 0.536, *η*^2^ = 0.130; DD: *F*_2,15_ = 0.765, *p*_corr_ = 0.483, *η*^2^ = 0.092).

## 4. Discussion

The temporal dissection of *foraging* expression in harvester ants reveals that the regulation of this gene is associated with worker behaviour at two time scales. On the scale of hours, gene expression undergoes changes greater than twofold, in magnitude, during the daily activity rhythms of foragers. On the scale of weeks to months, a shift in the daily temporal pattern of gene expression occurs during worker ontogeny from young workers inside the nest to older foragers. The temporal patterns of *foraging* gene expression in harvester ants are associated with worker task, age, location and exposure to light. Thus, our results reveal a complex gene × physiology × environment interaction, as would be expected for a behaviour-related gene that is one component of an intricate network. These associations are driven by significant fluctuations of *Pbfor* expression in workers of a particular task, foraging. Of the five worker groups studied, only foragers show significant daily fluctuations in *foraging* gene expression. The regulation of this gene in foragers is associated with daily activity patterns. Foragers have relatively higher expression during the day, when they are most active outside the nest, collecting food.

Our field results demonstrate an increase in *foraging* expression when foragers are most actively foraging outside the nest, exposed to light and other external environmental cues. However, nest maintenance workers and patrollers are also exposed to external conditions, although for a shorter amount of time than foragers, but these workers do not show significant diurnal changes in *foraging* expression. Foragers in laboratory colonies of a related species, *P. occidentalis,* also had high levels of *foraging* mRNA during the day [62]. This increase in *foraging* expression in harvester ant foragers may be associated with exposure to new external stimuli when the worker begins to forage, including exposure to light, and the rapid learning associated with foraging behaviour [44]. The fact that gene expression does not change significantly over time for either nest maintenance workers or patrollers in field colonies suggests that if external factors do indeed influence the *foraging* pathway, the duration of exposure to the external environment may be important.

Our experimental results support the hypothesis that exposure to light may modulate *foraging* expression in foragers but not brood workers. Expression levels of the *foraging* gene are depressed in foragers relative to brood workers in time points representing the dark phase of the LD treatment and are relatively low, and with more variability, in the DD treatment. Previous laboratory experiments on honeybees established a causal link between *foraging* gene expression, and foraging behaviour and suggested a potential role for *foraging* in phototaxis [34,35]. Differences in phototaxis between tasks have not yet been adequately tested in harvester ants. If *Pbfor* expression is linked to phototactic behaviour, then it is reasonable to expect that the levels of *Pbfor* in nest maintenance workers and patrollers would be similar to forager levels during their active hours outside the nest, but this was not the case in our study.

Alternatively, harvester ant foragers possess strong molecular circadian rhythms [61], and this internal clock may be linked to the regulation of behavioural genes involved in task allocation. The association of the circadian clock with the regulation of the *foragin*g gene gains support in this study from both behavioural and molecular data. In rhythmic foragers, the expression of *Pbfor* is correlated with daily behavioural patterns and with the expression of the clock gene, *cycle,* in both field and experimental conditions. Arrhythmic brood workers do not have differences in *foraging* expression with time of day, even when exposed to LD conditions. However, foragers typically maintain rhythms and cyclic expression of *cycle* in DD (data not shown), while we do not see significant differences in *foraging* expression under DD conditions in this study. These results suggest that *foraging* expression is modulated by the extended exposure to hours of light or other external factors, and is not simply influenced by endogenous rhythms. Another possibility is that significant oscillations in *foraging* expression are too difficult to measure given the weak circadian oscillations in DD conditions, a phenomenon also seen in some circadian genes.

Daily oscillations in the *foraging* gene were also evident in a microarray analysis of circadian rhythms in honeybee foragers, although subsequent qPCR analyses did not detect significant variation over time for either nurses or foragers [15]. The molecular pathways affected by circadian circuitry are not yet well understood, but recent work emphasizes multiple molecular responses to oscillations in circadian genes that are related to behaviour [15,70–73].

Our results highlight the importance of considering the effect of time on sampling expression levels of behavioural genes, particularly those that are likely to be closely linked to circadian rhythms. In harvester ants, daytime levels of *foraging* mRNA can be higher in foragers relative to other task groups, but at some times of day, foragers exhibit lower expression levels of *foraging* than other task groups. This finding has two implications. First, brood workers and callows have relatively greater expression during late evening and early morning hours. This explains why, in a previous study in which ants were sampled at dawn [39], *foraging* expression was lower in foragers relative to other task groups. Second, our results indicate that some of the reported experimental differences among species in the patterns of *foraging* expression may not represent distinct associations of *foraging* gene expression with species-specific behaviour. Instead, differences in the timing of sample collections may lead to the inadvertent capture of discrete snapshots of expression levels across the daily fluctuations in expression of the gene. Our review of the methods in previous studies of social insects [34–36,42,43,50,52] did not provide enough detail on the timing of sampling to determine how much this may have influenced the results.

One limitation of this field study is that it is not possible to completely disentangle the effects of age from the effects of location and/or task. Because ants probably perform nest maintenance and patrolling across a range of ages, and some may overlap with ages of foragers, experiments would be needed using similar-age cohorts that perform different tasks. This has been done in elegant laboratory experiments on social insects (e.g. [34,52]) but would be difficult to do in a natural field experiment.

Gene expression and the presence of *foraging* mRNA do not necessarily translate to protein activity differences *in vivo*. Future experiments should examine expression patterns in FOR protein in brains of foragers versus workers of other task groups. Additionally, *in situ* studies of RNA levels will elucidate whether the differential regulation of *foraging* is limited to particular brain areas in particular task groups, or during particular stages of behavioural maturation. A study of the ant *Pheidole pallidula* indicates that the spatial distribution of the *foraging* protein in the brain differs between minor and major workers of this species [43]. Thus, there may be changes in the location of *foraging*-sensitive regions of the brain involved in the transitions between tasks in harvester ant workers that could be determined by immunohistochemical analyses.

The flexibility in the regulation of *foraging* expression underscores the potential importance of this gene in the development of behavioural plasticity in social insect workers [45]. The *foraging* gene is highly conserved in the Hymenoptera, with little evidence for functional evolution in amino acid sequence [62]. It appears that gene regulation is the integral mechanism associated with behavioural plasticity, at least in harvester ants. The growing body of work showing that the amino acid-encoding sequences of many genes affecting social behaviour are highly conserved opens an exciting new direction in sociogenomics [2,3,74]—to understand when and where these conserved genes are active and how these differences play a role in organizing behaviour. Our results emphasize the importance of understanding how gene expression influences behaviour in natural field environments as well as in laboratory settings. Understanding the diversity of mechanisms by which conserved molecular pathways regulate behavioural plasticity in workers is a central issue in social insect biology and is critical to unravelling the molecular organization of social behaviour.

## Supplementary Material

Suppl. Table 1. Suppl. Table 2.

## Data accessibility

Primer information and datasets supporting this article are available on Dryad database (doi:10.5061/dryad.nj811) [75].
