# Supplementary material for: Context-dependent expression of the foraging gene in field colonies of ants: the interacting roles of age, environment and task
Source: Proc Biol Sci. 2016 Aug 31;283(1837):20160841. doi: 10.1098/rspb.2016.0841 (PMC5013789; doi:10.1098/rspb.2016.0841)
Supplement: Suppl. Table 1. Suppl. Table 2. [file rspb20160841supp1.docx]

**Suppl. Table 1. ANOVA results for gene expression changes over time by task.**

|  | df | F | p* | *eta*(η)^2^ |
| --- | --- | --- | --- | --- |
| Callows | 6, 17 | 1.575 | 0.215 | 0.357 |
| Brood Care | 6, 19 | 2.837 | 0.055 | 0.460 |
| Nest Maintenance | 6,20 | 0.654 | 0.687 | 0.164 |
| Patrollers | 6,20 | 0.953 | 0.481 | 0.222 |
| Foragers | 6,20 | 3.613 | 0.019* | 0.520 |

**Suppl. Table 2. Correlation analyses of *foraging* and *cycle* expression values in five tasks sampled in field study.**

| **Task** | **Pearson Correlation** | **p-value** |
| --- | --- | --- |
| *Forager* | 0.80 | 0.03* |
| *Patroller* | 0.62 | 0.14 |
| *Nest Maintenance* | 0.31 | 0.51 |
| *Brood Care Worker* | -0.13 | 0.77 |
| *Callow* | -0.11 | 0.82 |
